# Night-shift work and its association with metabolic syndrome

**DOI:** 10.1097/MD.0000000000043598

**Published:** 2025-08-01

**Authors:** Shih-Chieh Lin, Wei-Chung Yeh, Zhu-Xuan Liu, Hui-Fang Hsu, Jau-Yuan Chen

**Affiliations:** a Department of Family Medicine, Chang-Gung Memorial Hospital, Linkou Branch, Taoyuan, Taiwan; b Department of Family Medicine, Chang-Gung Memorial Hospital, Keelung Branch, Keelung City, Taiwan; c Powertech Technology Inc, Hsinchu, Taiwan; d College of Medicine, Chang-Gung University, Taoyuan, Taiwan; e Department of Health Management and General Practice, Xiamen Chang-Gung Hospital, Xiamen, China; f School of Medicine, National Tsing Hua University, Hsinchu, Taiwan.

**Keywords:** circadian system, health effects, metabolic syndrome, night shift, nonstandard work hours, shift work, work place

## Abstract

This study explores the correlation between metabolic syndrome and night-shift work in Taiwan by analyzing employees’ annual health check data (March 2019). More than 9000 records were enrolled. Through the analysis of the annual health check data of a semiconductor factory’s day-shift and night-shift workers’ metabolic syndrome indicators, the relationship between night-shift work and metabolic syndrome was identified. According to the definition of long-term night work labor by the Ministry of Labor of Taiwan, this study divides all employees of the factory into long-term night work and non-long-term night work employees. Abnormal waist circumference (WC) was defined as WC ≥ 90 cm in males and WC ≥ 80 cm in females. Abnormal blood pressure was defined as systolic blood pressure ≥ 130 mm Hg, diastolic blood pressure ≥ 85 mm Hg, or taking medication for hypertension. Abnormal fasting blood sugar was defined as ≥100 mg/dL or taking medication for diabetes. Elevated triglyceride (TG) was defined as TG ≥ 150 mg/dL or taking TG-lowering medication. Abnormal high-density lipoprotein (HDL) was defined as HDL < 40 mg/dL in males and HDL < 50 mg/dL in females. A total of 9322 workers from the factory were recruited: 4039 workers were night-shift employees, and 5283 workers were non-night-shift employees. Night work status increased the risk of poor blood pressure (systolic pressure + 3.1%, diastolic pressure + 1.7%) and elevated TG (+5.7%). Night-shift workers had a higher body mass index and abdominal circumference than day-shift workers. Night-shift workers had a 3.6% higher risk of obesity/overweight. Logistic regression analysis demonstrated that, after adjusting for age, sex, and body mass index, night-shift work was significantly associated with a higher risk of metabolic syndrome (odds ratio = 1.17, 95% confidence interval 1.02–1.35). This study supports the hypothesis that circadian rhythm disruption plays a role in the development of metabolic syndrome. It is recommended that business units implement targeted health promotion strategies, including regular health screening, nutrition guidance, onsite exercise support, and psychosocial well-being programs. Future research should include longitudinal designs and sleep quality assessments to clarify causality.

## 1. Introduction

To maintain the operation of the production line, shift or night-shift work is common in the manufacturing industry. There is a general perception among night workers that sleep deprivation leads to negative health consequences including obesity.^[1]^ Metabolic syndrome is defined by a constellation of interconnected physiological, biochemical, clinical, and metabolic factors that directly increases the risk of cardiovascular disease, type 2 diabetes mellitus, and all cause mortality.^[2]^

The relationship between shift work and metabolic syndrome has been found in previous studies.^[3,4]^ One study has showed that shift work increases the chance of obesity and insulin resistance, and leads to diabetes.^[5]^ Night-shift workers have poor beta cell function, resulting in higher blood glucose levels after meals.^[6]^ A study on nursing staff pointed out that compared with day-shift workers, night-shift workers consumed more total daily calories (2005 vs 1850 kcal), as well as higher amounts of fatty acids (77.9 vs 70.4 g), cholesterol (277 vs 258 mg), carbohydrates (266 vs 244 g), and sucrose (55.8 vs 48.6 g), which contributed to a higher obesity rate among night-shift workers.^[7]^ In Taiwan, a hospital workers study on the correlation between night-shift work and metabolic syndrome, has pointed out that shift work often causes abnormal work-rest patterns among staff, resulting in the occurrence of metabolic syndrome.^[8]^

Physiological studies suggest that night-shift work may contribute to metabolic syndrome by disrupting the circadian rhythm, which plays a key role in regulating glucose metabolism, insulin sensitivity, and hormonal balance. Such misalignment can lead to increased visceral fat, elevated blood pressure, and adverse lipid profiles, which are hallmarks of metabolic syndrome.^[9–11]^

Our study will explore the correlation between metabolic syndrome and night-shift work in a northern science and technology factory in Taiwan. The labor health inspection data of the factory, which has more than 9000 employees, were collected. These were large-scale local data with indicator significance. By analyzing the differences in the metabolic syndrome indicators of the factory’s day- and night-shift workers’ annual health checks, the association between night-shift work and metabolic syndrome is identified. These research results can be used as a reference for the implementation and updating of domestic occupational safety and health policies.

## 2. Methods

### 2.1. Study design and participants

This is a retrospective cross-sectional study. More than 9000 records were enrolled, from the annual health check data of a semiconductor factory’s workers in Northern Taiwan. The project was approved by the Chang Gung Medical Foundation Institutional Review Board (202001887A3). Participants provided informed consent for the use of their anonymized health examination data for research purposes.

### 2.2. Data collection and participant selection

**Figure 1.**
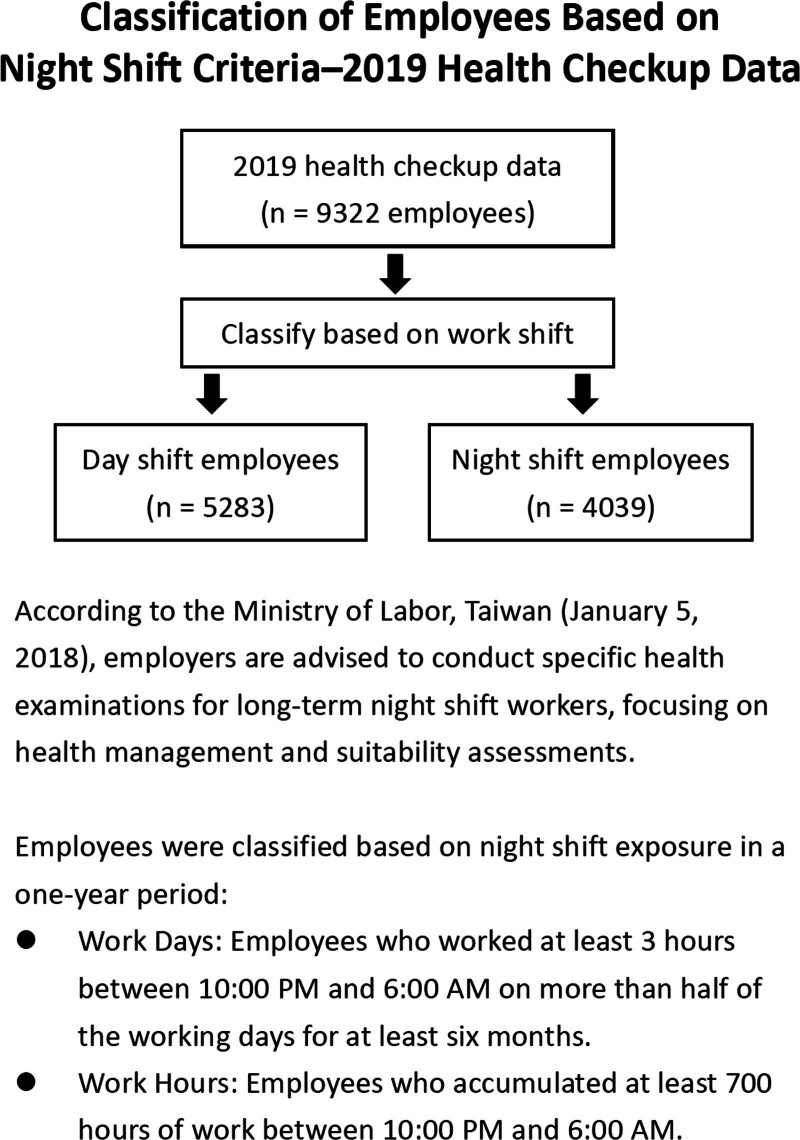
The data collection procedures.

The 2019 health examination dataset includes physical measurements and laboratory data collected from employees. The inclusion criterion was availability of complete metabolic syndrome indicators. Employees with incomplete or missing data were excluded. A total of 9322 employees were included in the analysis. As illustrated in Figure 1, participants were classified into day-shift and night-shift groups.

### 2.3. Definition of night-shift work

The Ministry of Labor of Taiwan defines long-term night workers in terms of working days and hours.^[12]^ Night work refers to work between 10 pm and 6 am If the total number of night work hours for a worker in the whole year is more than 700, or the worker works at night for at least 3 hours on 1/2 of the working days of the month for more than 6 months of the year, that worker is regarded as a long-term night worker. Those who meet the standard in 2018 will be subject to a health check in 2019.

### 2.4. Definition of metabolic syndrome

If 3 or more of the following 5 factors are met, the patient can be judged as having metabolic syndrome^[13,14]^: (1) abdominal obesity: male waist circumference ≥90 cm (35 inches) and female waist circumference ≥80 cm (31 inches); (2) high blood pressure: systolic blood pressure ≥130 mm Hg, diastolic blood pressure ≥85 mm Hg, or taking a doctor’s prescribed medicine for the treatment of hypertension; (3) fasting blood glucose level ≥100 mg/dL or taking medications prescribed by a doctor to treat diabetes; (4) fasting triglycerides ≥150 mg/dL or taking a doctor’s prescription for lowering triglycerides; (5) high-density lipoprotein cholesterol is low: males <40 mg/dL, females <50 mg/dL. This study used the above metabolic syndrome definition.

### 2.5. Confounding factors

Potential confounding factors such as age and sex were considered. These variables were adjusted for in both stratified analyses and logistic regression models to evaluate their impact on the association between night-shift work and metabolic syndrome.

### 2.6. Sample size considerations

No formal sample size calculation was performed. The study used existing health check data, and all available records were included in the analysis. A post hoc power analysis was conducted using G*Power. Assuming a 5% difference in outcome prevalence between night-shift and non-night-shift workers, the total sample size of 9322 provided over 90% statistical power at a significance level of 0.05. This is considered statistically and clinically reliable for observational research.

### 2.7. Statistical analysis

Descriptive statistics (mean, standard deviation, and percentage) were calculated for all baseline characteristics. Continuous variables were compared using independent two-sample *t* tests, and categorical variables were compared using chi-square tests. Logistic regression analysis was conducted to evaluate the association between night-shift work and metabolic syndrome, adjusting for age, sex, and body mass index (BMI). A *P*-value < .05 was considered statistically significant. All analyses were performed using SPSS.

## 3. Results

A total of 9322 people’s records employed by the factory in 2019 were collected, including those of 4039 long-term night workers and 5283 non-long-term night workers. Table 1 compares the continuous variables from the health checkup data related to day-shift and night-shift workers’ physiological status and metabolic syndrome. The analysis found that the night workers at the factory were relatively young, 1.9 years younger than day workers on average, and had 2 years less work experience than day workers on average. Night-shift workers also had higher BMI (+0.3 kg/m^2^), systolic blood pressure (+2.1 mm Hg), diastolic blood pressure (+0.9 mm Hg), waist circumference (+0.9 cm), and triglyceride levels (+13.6 mg/dL). High-density lipoprotein cholesterol and LDL-C levels were slightly lower in night-shift workers. The above differences were all statistically significant.

**Table 1:** A comparison of health examination results of night and non-night workers.

	Night workers	Non-night workers	Difference	*P*-value
	n = 4039	n = 5283		
	Mean ± SD	Mean ± SD		
Age (yr)	33.0 ± 5.7	34.9 ± 6.3	‐1.9	<.001
Body height (cm)	164.1 ± 8.7	164.9 ± 8.6	‐0.8	<.001
Body weight (kg)	68.1 ± 15.2	67.7 ± 15.1	0.4	.247
BMI (kg/m^2^)	25.1 ± 4.4	24.8 ± 4.4	0.3	<.001
SBP (mm Hg)	119.6 ± 13.3	117.5 ± 13.3	2.1	<.001
DBP (mm Hg)	78.2 ± 8.7	77.3 ± 8.5	0.9	<.001
Pulse	83.3 ± 11.1	83.2 ± 10.8	0.1	.895
WC (cm)	80.7 ± 11.2	79.8 ± 11.2	0.9	<.001
Cholesterol (mg/dL)	191.7 ± 34.7	192.6 ± 34.0	‐0.9	.180
TG (mg/dL)	142.4 ± 122.9	128.8 ± 114.8	13.6	<.001
FPG (mg/dL)	87.4 ± 24.9	88.8 ± 18.5	‐1.4	.003
HDL-C (mg/dL)	54.1 ± 14.1	55.7 ± 14.4	‐1.6	<.001
LDL-C (mg/dL)	110.9 ± 30.7	112.2 ± 30.3	‐1.3	.041

Clinical characteristics are expressed as mean ± SD for continuous variables. *P*-value were derived from independent two-sample *t* test for continuous variables.

BMI = body mass index, DBP = diastolic blood pressure, FPG = fasting plasma glucose, HDL-C = high-density lipoprotein cholesterol, LDL-C = low-density lipoprotein cholesterol, SBP = systolic blood pressure, TG = triglyceride, WC = waist circumference.

Table 2 compares categorical variables related to metabolic syndrome. Night-shift workers showed significantly higher rates of elevated blood pressure (+3.2%), triglycerides (+5.7%), and BMI over the overweight threshold (+3.6%). However, the prevalence of metabolic syndrome was only slightly higher (+1.2%) in night-shift workers and not statistically significant.

**Table 2:** A comparison of metabolic syndrome related health examination results of night and non-night workers.

	Night workers		Non-night workers		*P*-value
	n = 4039		n = 5283		
	n	%	n	%	
Age (yr)					<.001
<30	1518	37.6	1146	21.7	
30–39	2119	52.4	2935	55.5	
40–49	386	9.6	1114	21.1	
≥50	16	0.4	88	1.7	
Gender					<.001
Male	2169	53.7	2486	47.1	
Female	1870	46.3	2797	52.9	
BMI (kg/m^2^)					<.001
Underweight (<18.5)	118	2.9	217	4.1	
Healthy (≤18.5 to <24)	1727	42.8	2388	45.2	
Overweight (>24)	2194	54.3	2678	50.7	
SBP (mm Hg)					<.001
Normal (<130)	3190	79.0	4339	82.1	
Abnormal (≥130)	849	21.0	944	17.9	
DBP (mm Hg)					.038
Normal (<85)	3245	80.3	4334	82.0	
Abnormal (≥85)	794	19.7	949	18.0	
High blood pressure					<.001
Normal	2927	72.5	4001	75.7	
High (SBP ≥ 130 or DBP ≥ 85)	1112	27.5	1282	24.3	
WC (cm)					.137
Normal (M < 90 or F < 80)	2982	73.8	3972	75.2	
Abnormal (M ≥ 90 or F ≥ 80)	1057	26.2	1311	24.8	
TG (md/dL)					<.001
Normal (<150)	2752	68.1	3869	73.8	
Abnormal (≥150)	1287	31.9	1376	26.2	
FPG (mg/dL)					.177
Normal (<100)	3624	89.7	4694	88.9	
Abnormal (≥100)	415	10.3	589	11.1	
HDL-C (mg/dL)					.276
Normal	3209	79.5	4215	80.4	
Abnormal (M < 40 or F < 50)	830	20.5	1030	19.6	
Metabolic syndrome					.090
No	3448	85.4	4542	86.6	
Yes	591	14.6	703	13.4	
Cholesterol (mg/dL)					.167
Normal (<200)	2573	63.7	3268	62.3	
Abnormal (≥200)	1466	36.3	1977	37.7	

Clinical characteristics are expressed as n (%) for categorical variables. *P*-value were derived from independent chi-square test for categorical variables.

BMI = body mass index, DBP = diastolic blood pressure, FPG = fasting plasma glucose, HDL-C = high-density lipoprotein cholesterol, SBP = systolic blood pressure, TG = triglyceride, WC = waist circumference.

Table 3 shows the results of the age stratification analysis. After age stratification, the systolic blood pressure, diastolic blood pressure, and waist circumference of night-shift workers in each age group were all significantly higher. Workers aged 30 to 49 also had higher BMI and triglycerides. These results suggest that night-shift workers over 30 years old are more prone to obesity-related indicators.

**Table 3:** The comparison of clinical characteristics at night and non-night workers by age group.

	Age < 30			30–39			40–49			≥50		
	Night workers	Non-night workers		Night workers	Non-night workers		Night workers	Non-Night workers		Night workers	Non-Night workers	
	n = 1518	n = 1146		n = 2119	n = 2935		n = 386	n = 1114		n = 16	n = 88	
	Mean (SD)	Mean (SD)	*P*-value	Mean (SD)	Mean (SD)	*P*-value	Mean (SD)	Mean (SD)	*P*-value	Mean (SD)	Mean (SD)	*P*-value
Age (yr)	27.3 (2.2)	26.5 (2.1)	<.001	34.9 (2.7)	34.6 (2.8)	<.001	43.5 (2.5)	42.8 (2.5)	<.001	53.6 (1.9)	52.3 (3.0)	.086
Body height (cm)	162.8 (8.5)	163.8 (8.7)	.002	164.9 (8.7)	165.3 (8.7)	.066	165.4 (8.6)	165.1 (8.3)	.528	160.8 (7.9)	161.0 (7.7)	.932
Body weight (kg)	64.9 (15.2)	64.9 (14.6)	.956	69.6 (14.8)	68.5 (15.4)	.011	72.1 (14.9)	68.9 (14.5)	<.001	69.8 (15.8)	63.6 (13.6)	.108
BMI (kg/m^2^)	24.3 (4.4)	24.0 (4.3)	.088	25.5 (4.3)	24.9 (4.5)	<.001	26.3 (4.6)	25.1 (4.2)	<.001	26.8 (4.8)	24.4 (3.8)	.025
SBP (mm Hg)	116.5 (11.5)	114.8 (11.5)	<.001	120.6 (13.4)	117.0 (12.9)	<.001	125.2 (15.6)	120.9 (15.1)	<.001	134.6 (15.6)	123.8 (15.3)	.011
DBP (mm Hg)	75.9 (7.4)	75.3 (6.9)	.046	79.0 (8.9)	77.1 (8.2)	<.001	82.1 (10.2)	79.9 (10.1)	<.001	84.0 (8.4)	79.7 (8.9)	.076
WC (cm)	78.3 (11.1)	77.3 (11.0)	.030	81.7 (10.9)	80.3 (11.3)	<.001	84.4 (10.9)	81.0 (11.0)	<.001	85.2 (10.7)	79.4 (10.6)	.049
Cholesterol (mg/dL)	185.8 (33.0)	185.6 (32.9)	.872	194.2 (35.2)	193.4 (34.1)	.463	199.9 (35.5)	197.0 (33.6)	.151	215.7 (26.5)	200.9 (31.7)	.081
TG (mg/dL)	124.4 (102.5)	112.6 (98.4)	.003	149.4 (127.7)	130.9 (123.1)	<.001	174.1 (156.3)	138.5 (96.1)	<.001	141.1 (88.3)	148.0 (195.7)	.890
FPG (mg/dL)	84.0 (15.4)	85.8 (15.3)	.004	88.0 (24.8)	88.1 (17.9)	.882	97.1 (45.5)	93.2 (21.6)	.108	92.7 (11.2)	96.3 (22.2)	.531
HDL-C (mg/dL)	55.8 (14.1)	56.7 (14.0)	.093	53.3 (13.8)	55.7 (14.4)	<.001	51.9 (14.4)	54.5 (14.4)	.003	59.7 (19.8)	58.7 (15.4)	.823
LDL-C (mg/dL)	106.3 (29.2)	107.2 (29.1)	.431	113.1 (31.4)	112.7 (30.6)	.709	116.5 (31.0)	115.7 (30.0)	.652	128.3 (26.3)	115.9 (29.2)	.118

Clinical characteristics are expressed as mean ± SD for continuous variables. *P*-value were derived from independent two-sample *t* test for continuous variables.

BMI = body mass index, DBP = diastolic blood pressure, FPG = fasting plasma glucose, HDL-C = high-density lipoprotein cholesterol, LDL-C = low-density lipoprotein cholesterol, SBP = systolic blood pressure, TG = triglyceride, WC = waist circumference.

Table 4 summarizes the logistic regression results. In the unadjusted model, night-shift work was not significantly associated with metabolic syndrome (odds ratio = 1.12, 95% confidence interval = 0.99–1.26, *P* = .07). However, after adjusting for age, sex, and BMI, night-shift work became a significant predictor (odds ratio = 1.17, 95% confidence interval = 1.02–1.35, *P* = .02). Age, sex, and BMI were also independently associated with increased risk of metabolic syndrome.

**Table 4:** Logistic regression analysis for risk of metabolic syndrome.

Variables	OR	(95% CI)	*P* value	OR	(95% CI)	*P* value
	Unadjusted			Adjusted odds ratio		
Night worker (Ref: non-night worker)	1.12	(0.99–1.26)	.07	1.17	(1.02–1.35)	.02
BMI	1.35	(1.33–1.37)	<.001	1.34	(1.32–1.37)	<.001
Age (yr)	1.07	(1.06–1.08)	<.001	1.07	(1.06–1.09)	<.01
Male (Ref: female)	1.91	(1.70–2.17)	<.001	1.28	(1.12–1.48)	.001

Odds ratios and 95% confidence intervals were derived from binary logistic regression analyses to evaluate the association between night-shift work and metabolic syndrome. The adjusted model controlled for age, sex, and BMI.

Non-night-shift workers and females were used as reference groups. A *P*-value < .05 was considered statistically significant.

BMI = body mass index, CI = confidence interval, OR = odds ratio.

## 4. Discussion

Night-shift workers often encounter problems such as difficulty in exercising regularly and maintaining a normal diet. This study found that long-term night work may indeed lead to elevated blood pressure and an increased risk of obesity. The results indicated night-shift workers had higher rates of abnormal blood pressure (+3.2%), increased BMI (+3.6%), and elevated fasting triglycerides (+5.7%). These differences were statistically significant, and the same trends were observed across age stratifications.

Previous findings from large cohort analyses support these results, indicating that night-shift workers frequently present with elevated blood pressure, increased blood glucose levels, and abnormal lipid profiles, underscoring the importance of targeted health promotion efforts.^[15]^

Although many variables showed statistically significant differences between day and night workers, the actual differences between the 2 groups appeared minimal. It is possible that the large sample size increased the sensitivity of the statistical analysis, resulting in small differences achieving statistical significance.

Table 3 shows the results of the age stratification analysis. After age stratification, high blood pressure and waist circumference of night workers were observed in all age groups, and the differences were statistically significant. It can also be observed that night workers over the age of 30 had higher fasting triglycerides. Thus, night workers over 30 years old had a higher prevalence of obesity, which may have an adverse effect on their health. It is recommended that night workers over the age of 30 pay special attention to their risks of cardiovascular disease, obesity and fatty liver in the future and exercise more to maintain a healthy physiology.

After adjusting for age and sex in a logistic regression analysis, night-shift workers were found to have a significantly higher risk of developing metabolic syndrome. This indicates that age and sex may have masked the relationship in unadjusted analysis, and when controlled for, the occupational exposure to night work shows a clear association with metabolic syndrome. Similar findings have been reported in previous large cohort studies,^[16,17]^ where night-shift work remained significantly associated with metabolic syndrome even after controlling for major confounders.

One study examined the association between shift work and metabolic syndrome in 27,485 workers,^[18]^ finding that shift work may lead to obesity, high triglycerides and low high-density cholesterol, providing some evidence of the relationship between metabolic syndrome and shift work. Another systematic study pointed out that the occurrence of metabolic syndrome may be positively correlated with shift work, but perturbing factors such as sleep must be controlled.^[19]^ Besides, most studies have pointed out that in people working the night shift or shift work, the chance of metabolic syndrome increases and may even lead to cardiovascular disease, type 2 diabetes or stroke.^[3,19–23]^

These consistent findings across different populations may be explained by the shared biological mechanism of circadian rhythm disruption induced by night-shift work. Disruption of the circadian system impairs neuroendocrine pathways involved in feeding behavior and energy metabolism, leading to disturbances in glucose and lipid homeostasis.^[9]^ Furthermore, maintaining a physiological circadian rhythm is crucial for metabolic health, and its misalignment has been identified as a significant contributor to the development of metabolic diseases such as obesity and type 2 diabetes.^[24]^ These mechanisms provide a plausible explanation for the increased risk of metabolic syndrome observed among night-shift workers.

One study indicated that raising awareness among female shift workers may be a critical initial measure in reducing the risk of metabolic syndrome. In addition to awareness initiatives, complementary interventions such as dietary education, exercise counseling, and environmental support for physical activity are considered beneficial.^[25]^

Considering that night-shift workers may not easily implement a good exercise schedule, it is recommended that business units strengthen such efforts as building sports facilities on site and providing meals with a better nutritional balance and calorie content, in addition to providing more desirable food, through menu redesign.

In addition to physical health strategies, workplace health promotion programs that focus on psychosocial well-being, such as stress management, the prevention of workplace violence,^[26–28]^ and mindfulness-based interventions,^[29]^ may play an important role in alleviating emotional exhaustion and other psychological burdens commonly experienced by night-shift workers.

Furthermore, it is recommended that business units offer regular health screenings, particularly targeting long-term night-shift workers, to enhance their health awareness. Such initiatives should emphasize education on proper dietary habits, as well as active monitoring and management of blood pressure and body weight, in order to comprehensively improve their overall health status.^[30]^

This study is a cross-sectional data. Differences in labor age, gender, and work content do not deliberately exist in the selection of day and night labor. However, the statistical model adjustment suggests that age and sex may still confound the association in univariate comparisons. Previous literature also identified sleep status as a significant potential confounder in shift work research related to metabolic syndrome.^[31]^ It is recommended that similar studies in the future can be combined with sleep quality assessment to in-depth clarify the correlation between the occurrence of metabolic syndrome and shift work.

## 5. Strengths and limitations

This study benefits from a large sample size and comprehensive metabolic indicators, enhancing the robustness of its findings. Age stratification and adjustment for major confounders also strengthen the validity of the observed associations. However, the cross-sectional design limits causal inference, and factors such as sleep quality and dietary intake were not assessed.

## 6. Conclusion

Night-shift work is associated with a higher risk of metabolic syndrome, likely mediated by circadian disruption. Targeted health promotion strategies and workplace interventions are warranted. Future longitudinal studies should include sleep and lifestyle assessments to clarify causal pathways and guide prevention efforts.

## Acknowledgments

This research was successfully completed. Thanks to the colleagues in the factory and the industrial safety and health department who participated in the research. The health status of nighttime workers is valued by the competent authorities and institutions, and the occupational health research report is required by the labor health protection rules. The author is fortunate to complete a series of long-term night-work studies, which will be a local reference material for the health assessment of night-shift workers in the future. Thanks here.

## Author contributions

**Conceptualization:** Shih-Chieh Lin, Jau-Yuan Chen.

**Data curation:** Shih-Chieh Lin, Zhu-Xuan Liu, Hui-Fang Hsu.

**Formal analysis:** Zhu-Xuan Liu.

**Investigation:** Hui-Fang Hsu.

**Software:** Hui-Fang Hsu.

**Supervision:** Wei-Chung Yeh, Jau-Yuan Chen.

**Validation:** Shih-Chieh Lin, Wei-Chung Yeh.

**Visualization:** Shih-Chieh Lin, Jau-Yuan Chen.

**Writing – original draft:** Shih-Chieh Lin.

**Writing – review & editing:** Shih-Chieh Lin, Wei-Chung Yeh, Jau-Yuan Chen.
